# Effects of Different Ankle Supports on the Single-Leg Lateral Drop Landing Following Muscle Fatigue in Athletes with Functional Ankle Instability

**DOI:** 10.3390/ijerph17103438

**Published:** 2020-05-14

**Authors:** Cheng-Chieh Lin, Shing-Jye Chen, Wan-Chin Lee, Cheng-Feng Lin

**Affiliations:** 1 Department of Physical Therapy, Tzu Hui Institute of Technology, Pingtung 926001, Taiwan; carllin12@gmail.com; 2 Department of Product Design, College of Design, Tainan University of Technology, Tainan 71002, Taiwan; te0056@mail.tut.edu.tw; 3 Department of Physical Therapy, College of Medicine, National Cheng Kung University, Tainan 70101, Taiwan; onechie@gmail.com; 4 Institute of Allied Health Sciences, College of Medicine, National Cheng Kung University, Tainan 70101, Taiwan

**Keywords:** ankle instability, taping, fatigue, ground reaction force, single-leg landing

## Abstract

Background: Ankle support has been utilized for athletes with functional ankle instability (FAI), however, its effect on the landing performance during muscle fatigue is not well understood. This study aimed to examine the effects of ankle supports (ankle brace vs. Kinesio tape) on athletes with FAI following fatigued single-leg landing. Methods: Thirty-three young FAI athletes (CAIT scores < 24) were randomly allocated to control (Cn), ankle brace (AB) and Kinesio tape (KT) groups. All athletes performed single-leg lateral drop landings following ankle fatigue protocol. The fatigue-induced changes in kinetic parameters were measured among three groups. Results: A significant increase in peak vertical ground reaction force (vGRF) was found in the AB group (0.12% body weight (BW)) compared to that of the KT (0.02% BW) and Cn (median = 0.01% BW) groups. Significant decrease in both COP medial-lateral (ML) and anterior-posterior (AP) ranges were also found in the KT group (median = −0.15% foot width (FW) & median = −0.28% foot length (FL)) than those of the Cn group (median = 0.67% FW& median = 0.88% FL). Conclusions: Ankle braces might hamper the ability to absorb the impact force during landing. On the other hand, Kinesio tape might be beneficial for the postural control during landing.

## 1. Introduction

Ankle sprain occurs frequently in the sports population and may result in varying degrees of debilitation [1,2]. Functional ankle instability (FAI) is described as a condition in which athletes may report sensations of “giving way” at their ankles and experience recurrent ankle sprains [3]. Ligamentous injuries of the ankle resulted from the FAI often produce proprioceptive deficits of the injury leg, which would have a negative effect on postural control during performance [4,5,6,7,8].

Many ankle sprains occur on landing during sports activity with or without contact [9]. Alterations in landing performance have been observed in athletes with FAI, and these alterations may potentially increase the risk for recurrent injury, and ankle joint degeneration [6,9,10,11,12]. For example, Wikstrom et al. examined the dynamic postural control of a single-leg-hop task, and found reduced stability in the anterior and posterior and vertical direction in individuals with FAI compared to individuals with stable ankles [6]. Furthermore, fatigue may play a significant role in the occurrence of ankle injuries, and many injuries occur during the latter stages of matching or training periods when fatigue is present [13,14,15,16]. Various biomechanical alterations following fatigue including an increase in peak proximal tibial anterior shear force, peak knee valgus angle, and knee internal rotation, and a decrease in knee flexion angle, hip flexion angle during landing, have been reported [17]. Fatigue has also been found to adversely affect the ankle proprioception, and the postural control [14,15,16]. Hence, athletes with FAI may be prone to a high risk of being injured when the lower extremities are fatigued. In addition, an increase of up to three to six times of individual’s body weights in peaked vertical ground reaction force (vGRF) has been observed in jump-landings in athletes with FAI, and the vGRF would be a valuable parameter for studies investigating the potential reduction mechanism of injuries [18].

External ankle support such as ankle braces, and taping is common preventive method for ankle sprain. Ankle braces may reduce injury by limiting excessive ankle inversion, and meta-analysis has demonstrated its effect in the prevention of ankle sprain [19,20]. Ankle taping has also been shown to effectively result in a two- to four-fold reduction in ankle sprain incidence, especially in those with ankle sprain history [19,20]. In addition, Kinesio tape has been proposed as an alternative to the bracing and taping techniques by providing support and stability to muscles and joints without restricting the body range of motion [21,22,23,24]. However, little quality evidence to support the use of kiesio tape over other types of taping in injury prevention are available [25,26].

Landing occurs in a wide variety of sports, and ligamentous damage, ankle muscle strength deficits, delayed muscle reaction time, and proprioception deficits resulted from the ankle sprain may cause athletes with FAI to land differently compared to those with stable ankles [10]. Furthermore, the alterations in the kinetic variables of the lower extremities such as vGRF have been consistently reported after fatigue [17]. However, the effect of ankle support on landing performance after fatigue remains unclear. The purpose of the present study is to investigate the effects of two common ankle support including ankle brace and Kinesio tape on landing performance after fatigue in athletes with FAI.

## 2. Materials and Methods

### 2.1. Participants

Athletes (*N* = 33) of 18~35 years old with functional ankle instability (FAI) were recruited in the study. The athletes were randomly allocated to a non-external support control group (8M3F, age: 22.0 ± 2.8 y), a lace-up ankle brace (AB) group (8M3F, age: 22.5 ± 1.9 y), and a Kinesio tape (KT) group (9M2F, age: 21.6 ± 3.0 y). To identify the individuals with FAI, the inclusion criteria were specified based on the guidelines proposed by Gribble et al. and Delahunt et al. as the following: (1) participating in a sports team and performing regular exercise three times a week for period of at least 30 min each time; (2) experiencing at least one significant ankle sprain within the past year that resulted in pain, swelling and the need to rest for a few days; (3) having residual symptoms such as episodes of giving way, pain, instability or weakness; (4) having a Cumberland Ankle Instability Tool (CAIT) score of less than 24 (for a score of less than 24 bilaterally, the ankle with the lower score was selected as the involved ankle); and (5) a negative anterior drawer test and talar tilt test result [27,28,29]. The exclusion criteria were: (1) acute ankle sprain consisting of swelling or inflammation symptoms; (2) a history of lower extremity fracture, surgery, or congenital bony deficit; and (3) existing neurological disorder or heart disease. Prior to participation, each subject signed the informed consent form approved by the Institutional Review Board of the affiliated University Hospital, and was informed of the experimental procedure.

### 2.2. Experimental Procedure

**Figure 1:**
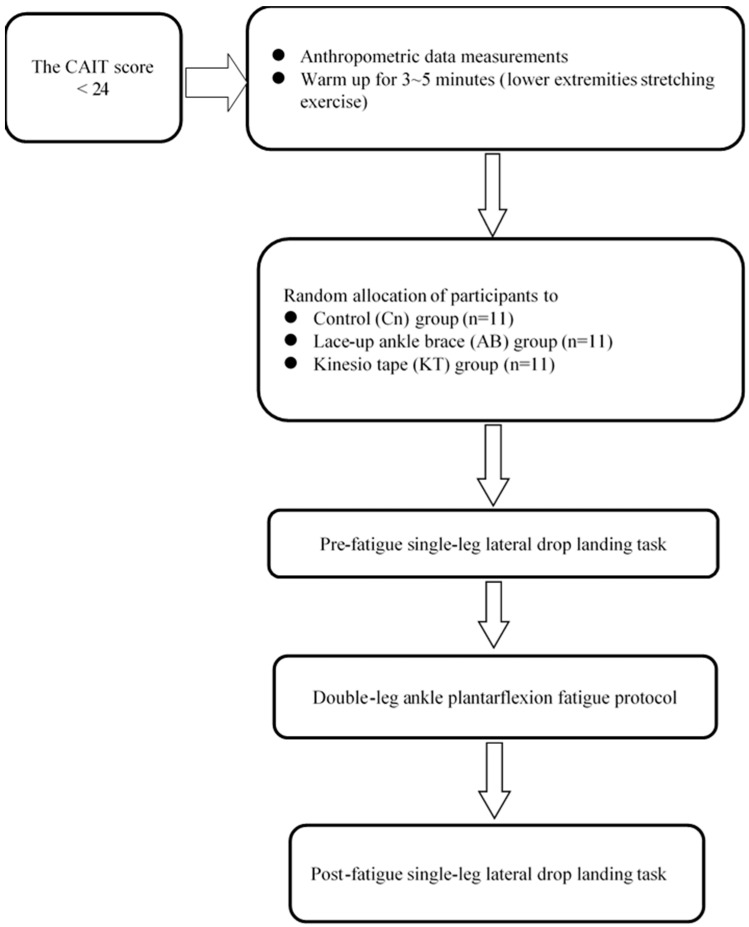
Flow chart of experimental procedure for Control (Cn), Ankle Brace (AB) and kinesio tape (KT) groups.

**Figure 2:**
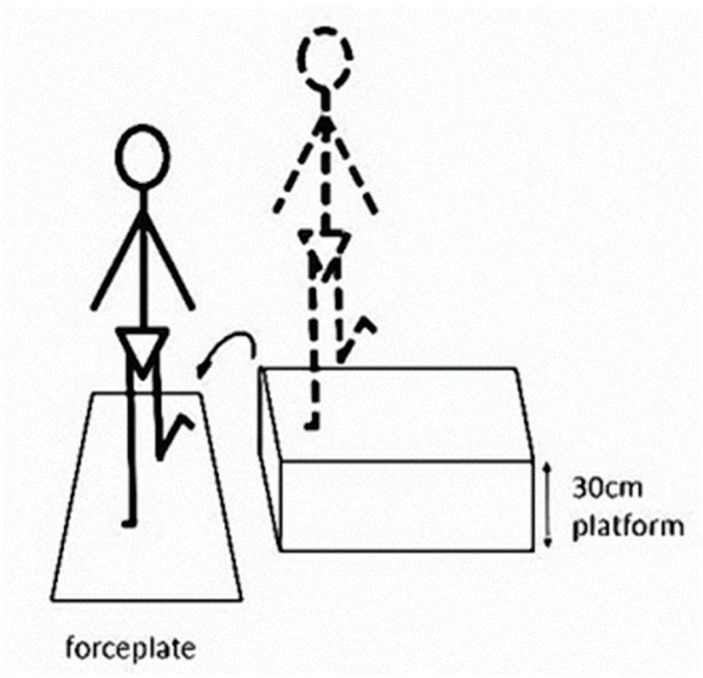
Schematic illustration of single-leg lateral drop landing task.

The flow chart of the experimental procedure was illustrated in Figure 1. Subjects wore standardized shoes and performed the single-leg lateral drop landing both before and after the fatiguing protocol with the involved leg. Subjects were instructed to stand on the involved leg with the knee extended on a platform with a height of 30 cm (Figure 2), and maintain balance for 5 s and then to perform a lateral drop landing on the involved leg on a ground force plate. Following landing, subjects were instructed to regain stability as soon as possible and to maintain the trunk in an upright and forward-facing direction for at least 5 s. Practices were allowed for subjects to feel comfortable with the task, and a rest period could be taken before the beginning of the data collection. Three successful landing trials were collected both before and after the fatigue protocol. A rest period of 30 s was allowed between each trial. Trials in which the participants landed with foot wobbling were discarded. The ground reaction force (GRF) during landing was measured using a Kistler force plate (Kistler Instrument Corp., Winterhur, Switzerland) with a sampling frequency of 1000 Hz.

Fatigue was induced by asking subjects to stand on a footstool with their heels aligned in space, and place both hands slightly forward and against a wall to maintain balance. Subjects were asked to perform maximal ankle plantar flexion with the knee extended. The maximal heel height rise (as indicated by a heel reflective marker) was measured and recorded by the conductor. Subjects were then instructed to perform ankle plantar flexion consecutively and rhythmically at a rate of one time per second (paced by a metronome). Verbal encouragement was provided, and subjects were reminded to perform ankle plantar flexion to the maximal extent possible. The fatigue protocol was performed until the subjects failed to reach 70% of the maximal heel height rise on three consecutive attempts [30]. Immediately following the fatigue protocol, subjects were asked to rate the subjective feeling of exertion using the Borg Rating of Perceived Exertion (RPE) [31]. The number of heel-raising trials to attain fatigue in each group was 382.9 ± 98, 468 ± 132, 468 ± 109 for Cn, AB, and KT group, respectively, with no significant difference between groups.

**Figure 3:**
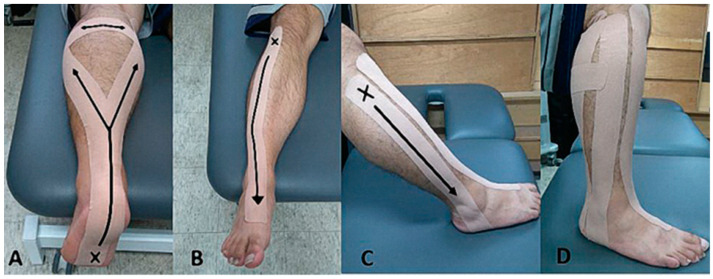
Application of kinesio tape to muscle groups of involved leg: (**A**) gastrocnemius; (**B**) tibialis anterior; (**C**) peroneal longus; and (**D**) overall view. Note that “x” indicates the origin anchor of the kinesio tape and the arrow shows the direction in which the tape is applied.

A lace-up rigid ankle brace (Mueller Sports Medicine, Inc., Prairie du Sac, WI, USA) and Kinesio tape (SKT-X-050R, Nitto Denko Corp., Ibaraki, Osaka, Japan) was applied on the involved leg of the AB and KT group, respectively, in the fatigue protocol and post-fatigue tasks. The ankle was plantar flexed and everted while the tape was applied on the tibialis anterior, and was dorsi flexed and inverted while the tape was applied on the peroneal longus for the purpose of muscle facilitation [32,33]. The tape was also applied on the gastrocnemius from muscle insertion to the origin to prevent muscle over-contraction (Figure 3). Subjects were asked to follow the given instructions carefully and the conductor placed the tape on each designated muscle group without tension.

### 2.3. Data Analysis

The GRF data were processed using a self-written algorithm coded via MATLAB (Version R2012b, Mathworks Inc., Natick, MA, USA). The vGRF was measured after landing and was normalized by the body weight. The loading time was defined as the moment of initial contact to the point of peak vGRF. The average loading rate was then computed as the peak vGRF divided by the loading time. The center of pressure (COP) range and velocity were analyzed in both the anterior–posterior (AP) direction and the medial–lateral (ML) direction and were normalized by the foot length and foot width, respectively. Statistical analyses were performed using SPSS18.0 software (SPSS for Windows, Chicago, IL, USA). *P* < 0.05 was considered significant in all tests. The differences between groups were examined using the nonparametric statistics Kruskal–Wallis one-way analysis of variance by ranks test because the number of subjects in each group was small, and not normally distributed.

Specifically, the values of all the dependent variables were expressed as a “difference” in values obtained before and after the fatigue protocol. The “difference” values of the dependent variables in each group was then compared using the Kruskal–Wallis method in order to determine the significance of the median values of the dependent variables among the three groups. When significant main group effect was found, post-hoc tests were carried out.

## 3. Results

The athletes recruited in the present study participated in various team sports, including basketball, volleyball, baseball, and badminton. All of the athletes had a similar degree of FAI with the CAIT score in the range of 16~18 (Table 1). The average RPE score reported by the athletes was 18 for the fatigue protocol, indicating a hard to extremely hard degree of exertion.

Significant difference between groups was found for the median value of the difference in peak vGRF before and after the fatigue protocol with biggest value in the AB group followed by the KT and Cn group (Table 2). Moreover, the post hoc analyses showed that the difference between the AB group and the other two group reach the significance (*p* = 0.019 for AB vs. Cn group, and *p* = 0.049 for AB vs. KT group) (Table 2).

No significant group difference was found for either the difference of the loading rate or loading time before and after the fatigue protocol (*p* = 0.053 and 0.139 for the loading rate and loading time) (Table 2).

Regarding the range of the COP in both AP and ML direction, the value obtained after the fatigue protocol was smaller in the KT group but was larger in the Cn and AB group compared to the value obtained before the fatigue protocol (Table 3). The post hoc test showed that the KT group had a significantly smaller median difference in the COP range than the Cn group (*p* = 0.028) and AB group (*p* = 0.039) in the ML direction. Similarly, the median value of the difference of the COP range was also significantly smaller in the KT group than the Cn group (*p* = 0.014). Moreover, the median value of the difference in the COP velocity was also significantly smaller than the Cn group (*p* =.014) and AB group (*p* = 0.02) (Table 3).

## 4. Discussion

The present study aimed to understand the effects of external support (ankle brace (AB) and Kinesio tape (KT)) on the kinetic change induced by ankle plantar flexor muscle fatigue in athletes with FAI in single-leg lateral landing. The AB group was found to exhibit a significantly greater change in the peak vGRF following fatigue than the KT group and Cn group. In addition, the KT group showed a smaller range of the postural sway following landing than the AB group or Cn group.

In the present study, the AB group exhibited the largest changes in the peak vGRF of the three groups during landing. External ankle supports are designed to protect the ankle from injury by restricting frontal plane motion. During a jump landing task, joint motions begin distally and progress proximally for the purpose of energy dissipation [34,35]. The entire lower extremity may be affected, and the changes in movement patterns may occur when the normal motion or the muscular function around the ankle is restricted. The restriction of the ankle motion by the ankle brace may hamper the ability of the lower extremity to attenuate vGRF [36,37,38,39]. The vGRF may be as high as 6.2 times body weight during a jump landing task [40], and hence, the reduced ability to dissipate force may also result in the increased risk of other lower extremity injuries. The increase in peak vGRF in AB group could be the result of reduced ankle joint motion due to the passive restraining effect of the brace [41,42,43]. On the other hand, the studies investigating effect of the Kinesio tape on the ground reaction force during landing remains scarce. Jafarnezhadgero et al. reported that athletes with concurrent patella–femoral pain syndrome and pronated feet displayed a lower peak posterior GRF amplitude while performing the bilateral drop landing tests with Kinesio tape compared to the no-tape condition [44]. Ho et al. also observed the deceased vertical GRF for athletes with FAI performing vertical jump after taping [45]. Contrary to these above-mentioned findings, no significant difference was observed in the change of the peak vGRF between the KT group and the control group. The possible reason for the discrepancy in study results may include the difference in task (bilateral drop landing vs. single-leg lateral landing), taping methods and locations.

The loading rate and loading time are related to the shock wave transmitted through the body, and suggested to be associated with impact-related injuries [46,47,48,49]. A soft landing will allow deceleration to occur more slowly, thereby preventing structures from the higher forces associated with rapid deceleration [50,51]. Studies have shown that the GRF loading time is shortened in subjects with FAI performing jump landing tasks than healthy individuals [18,52]. Riemann et al. demonstrated that the time to reach peak vGRF was significantly less and the peak vertical impact forces were greater during drop landings with the ankle braces and tape conditions [37]. Cerullo et al. also observed the decreased time to absorb rearfoot impact force during landing with the combination of ankle and spat taping [53]. The increased loading rate or the decreased loading time may impede the ankle′s ability to act as an efficient shock absorber, and thus transmit the impact force to the proximal joints of the lower extremity. Despite no statistical significance, the values of the difference in the loading rate before and after the fatigue protocol were bigger in the AB and KT group than the Cn group, suggesting that both the application of the ankle brace and Kinesio tape may have affected the shock absorption during landing.

FAI may result from the proprioception deficits in ankle ligaments due to the damage of ankle ligament sensory receptors, leading to a diminished supply of messages related to joint movement and position to afferent pathways. Ross and Guskiewicz reported impaired postural control in subjects with FAI by observing a significantly longer time for stabilization in subjects with FAI than subjects with stable ankles after a single-leg jump [10]. However, the effect of the ankle brace and Kinesio tape on proprioception remains unclear. Feuerbach et al. reported that the variable and absolute error in matching the reference positions were significantly less with the brace than without, and proposed that the brace application may increase the afferent feedback from the cutaneous receptors, leading to an improved ankle proprioception [54]. However, a meta-analysis concluded that the ankle brace or ankle tape had no effect on proprioceptive activity in subjects with FAI [55]. In the present study, the COP range decreased in the KT group, but increased in the Cn and AB group in both the AP and ML direction, with significant differences between the KT group and Cn group in AP direction, and between the KT group and Cn and AB group in ML direction. To date, inconsistencies remain regarding the effect of ankle brace or Kinesio tape on the postural control. Several studies have shown the limited effect of ankle brace on dynamic stability due to the reduction in the ankle range of motion [56,57,58]. However, Shaw et al. have found that ankle braces have a positive effect on postural control in fatigue condition in volleyball players [59]. Hadadi et al. also reported a decrease in COP parameters with the use of an ankle brace during quiet standing [59]. A number of studies evaluating the effect of Kinesio tape on postural control via the Star Excursion Balance Test have consistently found no improvement [21,60,61,62,63]. Other studies evaluating postural sway during quiet standing or single-legged balance tests have demonstrated more promising results [24,64,65,66]. Differences in studied population, outcome variables, type of braces, and taping method make the comparison among studies difficult. Our findings provide the evidence regarding the effect of the Kinesio tape on the postural control during the single-leg lateral drop landing task. The physiological mechanism by which the Kinesio tape may have been presumed to work remains unclear. The improvement of the postural control during landing may be achieved by the improved proprioception around the ankle resulting from increased cutaneous afferent feedback from tape application [67,68,69].

Several limitations should be acknowledged in the present study. First, the number of subjects included in each group was relatively small, and therefore, the current results must be interpreted with caution against type II statistical error. Second, the recruited athletes were not specialized in the same sport. Thus, their performance of the lateral drop landing task was inevitably affected to some degree by their particular sport type and skills. Third, the external ankle support including brace and Kinesio tape were only applied to the subjects for several hours, and hence, its effect in the long term and follow up needs further investigation. Fourth, no range of motion or muscle activity was recorded during landing in the present study. The significant role of the ankle plantarflexors has been demonstrated by showing the decrease in both peak vGRF and loading rate with the increase in the angle of plantar flexion during weight acceptance in landing 69]. As a result, the combination of ground reaction force, joint range of motion, and muscle activity measure will provide us better understanding of how external ankle support affects the landing performance in subjects with FAI.

## 5. Conclusions

In conclusion, the present study investigated the effects of lace-up ankle braces and Kinesio tape on the kinetics of FAI athletes performing lateral jump landing tasks in pre- and post-fatigue conditions. The results show that the ankle brace might inhibit the ability of the ankle joint to dissipate the impact force, and thus predisposes the athletes to secondary injuries. By contrast, the Kinesio tape may provide a better postural control for dynamic landing of later matches or training exercise.

## Figures and Tables

**Table 1:** Anthropometric data (Mean± standard deviation) and functional ankle instability evaluation scores.

	Control (*n* = 11)	Ankle Brace (*n* = 11)	Kinesiotape (*n* = 11)	*p* Value
Gender	8M3F	8M3F	9M2F	
Body height (cm)	173.6 ± 7.2	172.6 ± 9.1	171.2 ± 6.5	0.760
Body weight (kg)	65.4 ± 6.8	70.1 ± 16.9	63.5 ± 7.7	0.393
Age (yr)	22.0 ± 2.8	22.5 ± 1.9	21.6 ± 3.0	0.724
Duration of regular exercise (hr/week)	10.0 ± 7.4	7.3 ± 2.9	9.0 ± 4.0	0.498
CAIT score	16.3 ± 5.9	16.7 ± 3.4	18.2 ± 4.9	0.632
Post-fatigue RPE score	18.0 ± 1.0	17.9 ± 0.8	17.8 ± 1.7	0.828

CAIT: cumberland ankle instability tool; RPE score: rate of perceive exertion score.

**Table 2:** Kruskal–Wallis and post hoc test results for fatigue-induced change in median values of the kinetic variables in single-leg lateral drop landing tasks performed by three groups.

	Control	Ankle Brace	Kinesio Tape	K-W Test
Peak vGRF (%BW)				0.043 *
Pre-fatigue	3.20 ± 0.33	3.18 ± 0.35	3.38 ± 0.28	
Post-fatigue	3.19 ± 0.33	3.32 ± 0.40	3.34 ± 0.33	
Difference	−0.01	0.14 *^a,^*^b^	−0.05	
Loading rate (N/ms)				
Pre-fatigue	16.60 ± 2.71	17.90 ± 4.71	16.92 ± 2.31	
Post-fatigue	17.19 ± 2.70	19.78 ± 4.54	18.13 ± 2.52	0.053
Difference	0.59	1.88	1.21	
Loading time (ms)				
Pre-fatigue	123.81 ± 4.97	122.91 ± 9.57	124.64 ± 8.55	
Post-fatigue	118.97 ± 5.17	114.36 ± 8.69	114.42 ± 7.33	0.139
Difference	−4.84	−8.55	−10.21	

*: Significant difference; vGRF: vertical ground reaction force; BW: body weight; *a: A significant difference of peak vertical ground reaction force in ankle brace group greater than control group (*p* = 0.019); *^b^: A significant difference in peak vertical ground reaction force in ankle brace group greater than KT group (*p* = 0.049).

**Table 3:** Kruskal–Wallis and post hoc test results for fatigue-induced change in median values of the COP variables in single-leg lateral drop landing tasks performed by three groups.

	Control	Ankle Brace	Kinesio Tape	K-W Test
Difference of COP range				
ML (%FW)	0.67	0.77	−0.15 *^a,^*^b^	0.046 *
AP (%FL) Difference of COP velocity	0.88	0.57	−0.28 *^c^	0.039 *
ML (%FW/sec)	3.83	2.16	2.27	0.808
AP (%FL/sec)	−0.55	3.70	−4.50 *^d,^*^e^	0.018 *

*: Significant difference; ML: medial–lateral; AP: anterior–posterior; FW: foot width; FL: foot length; *a: A significant smaller difference of COP ML range in KT group than control group (*p* = 0.028); *b: A significant smaller difference of COP ML range in KT group than ankle brace group (*p* = 0.039); *c: A significant smaller difference of COP AP range in KT group than control group (*p* = 0.014); *d: A significant smaller difference of COP AP velocity in KT group than control group (*p* = 0.014); *e: A significant smaller difference of COP AP velocity in KT group than ankle brace group (*p* = 0.02); %FW: percentage of foot width; % FL: percentage of foot length; %FW/sec: percentage of foot width per second; %FL/sec: percentage of foot length per second.
